# 963. Establishing Clinical Benchmarks for Dermal Matrices in Full-thickness Management: Analysis of Role in Complex Closure

**DOI:** 10.1093/jbcr/irag033.541

**Published:** 2026-04-06

**Authors:** Roselle E Crombie, Derek Bell

**Affiliations:** Connecticut Burn Center/Yale New Haven Health, Westport, CT; University of Rochester Medical Center, Rochester, NY

## Abstract

**Introduction:**

Dermal matrices are used in full-thickness wound management to optimize wound bed preparation for autografting. These products promote vascularization, enhance autograft take, create an interface between scar and underlying structures ultimately improving long-term functional and aesthetic outcomes. However, the time required to prepare a wound for autografting can contribute to delayed healing, extend hospital stays, and increase resource utilization. Comparative evidence across products is limited, leaving clinicians without clear benchmarks for expected performance. This study aims to systematically review and synthesize published clinical evidence on commercially available dermal matrices to identify key endpoints and define performance goals that may guide future clinical research and value.

**Methods:**

A systematic literature review was conducted using PubMed/MEDLINE and Embase for studies published between January 2019 and October 2024. Benchmark products included collagen glycosaminoglycan, fish skin-derived, biodegradable temporizing, biodegradable polyurethane, and collagen elastin dermal matrices. Inclusion criteria included study designs using a 2-stage operative approach, discussion of safety and performance outcomes in human subjects, and publication in a peer-reviewed journal or book. Case reports, narrative reviews without outcome data, conference abstracts, and duplicate publications were excluded. A meta-analysis was then performed to establish pooled performance benchmarks for time to autografting, rate of wound closure, and time to healing.

**Results:**

Twenty-four publications met inclusion criteria, of which 18 contained sufficient data for quantitative analysis. The primary performance endpoint, time to autografting, resulted in a pooled mean of 31.8 days (95% CI 27.2-36.4 days). Wound closure (reported in n = 14 studies) was achieved in 95.8% of included studies (95% CI 86.9%-98.7%), with time to complete healing (reported in n = 8 studies) averaging 54.6 days (95% CI 33.8-75.4 days).

**Conclusions:**

Dermal matrices demonstrate consistent performance across published studies, but current benchmarks show a prolonged interval to autografting of approximately one month. These findings underscore the limitations of existing products and highlight the need for novel dermal matrices that can shorten time to definitive closure.

**Applicability of Research to Practice:**

In a resource restrictive environment, it's important to understand impact and begin to develop evidenced based guidelines for use.

**Funding for the study:**

N/A.

